# 

**Published:** 1858-12

**Authors:** 


### CONTENTS.

ORIGINAL ESSAYS AND COMMUNICATIONS.

Filling Teeth, by J. Taft,............................................................ 125
Continuous Gum Work, by Dr. W. B. Roberts,............................................ 130
Introductory Lecture, to the Dental Class of 1858-9, by Prof. C. B. Chapman,...... 148
Bonsall's Address,............................................................ 165
Signs of Lesions of Nerve Centers, by Prof. C. B. Chapman,.......................... 170
A Synopsis--Dental Ethics, by J. Taft,.............................................. 173
Dental Quacks,............................................................ 179
Porcelain Teeth, by Daniel L. Pratt,................................................ 185
Splitting of the Alveolar Process of the Lower Jaw,................................ 187
How to fix your Forceps before Extracting Teeth,.................................... 189
Kellum's Soldering Flask,............................................................ 190

SELECTIONS.

How Does Chloroform Cause Death, by T. L. Maddin, M. D.,.......................... 192
Remarks on Leeches, by Frederick Stearns,........................................... 201
How Teeth are Destroyed,............................................................ 203
Criminal Assault by a Dentist,....................................................... 212
Chloroform,............................................................ 230
Treatment of Fractures of the Lower Jaw, by W. W. Allport,........................ 230
Miscellaneous,............................................................ 232
Editorial............................................................ 239

